# Retrieval analysis of Harris-Galante I and II acetabular liners in situ for more than 10 years

**DOI:** 10.3109/17453674.2012.717843

**Published:** 2012-08-25

**Authors:** Keisha French, Rebecca Moore, Heather Gawel, Steven M Kurtz, Matthew J Kraay, Ke Xie, Victor M Goldberg, Clare M Rimnac

**Affiliations:** ^1^State University of New York Medical Center, New York, NY; ^2^Orthopaedic Implant Retrieval Analysis Laboratory, Department of Orthopaedics, Case Western Reserve University, Cleveland, OH; ^3^Implant Research Center, School of Biomedical Engineering, Science, and Health Systems, Drexel University and Exponent Inc., Philadelphia, PA; ^4^Departments of Mechanical and Aerospace Engineering and Orthopaedics, Case Western Reserve University, Cleveland, OH, USA

## Abstract

**Background and purpose:**

There have been few reports documenting the wear and oxidation performance of the polyethylene bearing surface of HGPI and HGPII THA devices.

We evaluated retrieved HGPI and HGPII acetabular liners that had been in situ for more than 10 years and determined whether there was a relationship between clinical and radiographic factors, surface damage, wear, and oxidation.

**Materials and methods:**

129 HGPI and II acetabular liners with implantation times of > 10 years were retrieved at 4 institutions between 1997 and 2010. The liners were made from a single resin and were gamma radiation-sterilized in air. Surface damage, linear wear, and oxidation index (OI) were assessed. Differences in clinical and radiographic factors, surface damage, linear wear, and OI for the 2 designs were statistically evaluated separately and together.

**Results:**

Articular surface damage and backside damage was similar in the 2 designs. The linear penetration rate was 0.14 (SD 0.07) mm/year for the HGPI liners and 0.12 (SD 0.08) mm/year for the HGPII liners. For both cohorts, the rim had a higher OI than the articular surface. 74% of the liners had subsurface cracking and 24% had a complete fracture through the acetabular rim.

**Interpretation:**

Despite modification of the HGP locking mechanism in the HGPII design, dissociation of the liner from the acetabular shell can still occur if fracture of the rim of the liner develops due to oxidative degradation.

Biological fixation of porous-coated cementless acetabular components has proven to be a reliable and durable method of implant fixation in total hip arthroplasty (THA) (Judet et al. 1978, Lord et al. 1979, Cruz-Pardos and Garcia-Cimbrelo 2001). Successful cementless fixation strategies for the acetabulum have included porous surfaces with sintered beads, plasma-sprayed titanium, or titanium fibermetal. Generally, these implant designs have incorporated screws, pegs, fins, or spikes to provide adjunctive fixation until tissue ingrowth into these cementless devices occurs.

The Harris-Galante Prosthesis I (HGPI) acetabular component (Zimmer, Warsaw, IN) was a hemispherical cementless acetabular component with a titanium fibermetal ingrowth surface and multiple holes to allow for supplementary screw fixation (Clohisy and Harris 1999). The HGPI and its successor, the HGPII, were two of the most widely used cementless acetabular components in the 1980s and 1990s. Both implant designs incorporated a modular acetabular bearing or liner made of ultra-high molecular weight polyethylene (hereafter called polyethylene). Modifications to the initial HGPI design were minor and consisted of alteration of the locking mechanism for the modular polyethylene liner (Clohisy and Harris 1999, Parvizi et al. 2004), a slight increase in the thickness of the hemispherical shell, and enlargement of the holes in the shell to allow for fixation with 6.5-mm screws as opposed to the 5.1-mm screws used with the HGPI design (Clohisy and Harris 1999, Parvizi et al. 2004).

At the time that these implants were available for clinical use, the polyethylene liners were all sterilized by gamma irradiation in air. Several studies have shown that these designs have performed well (Schmalzried and Harris 1992, Berger et al. 1997, Tompkins et al. 1997, Cruz-Pardos and Garcia-Cimbrelo 2001) with regard to fixation and osseointegration in both primary THA and the challenging setting of revision THA with significant acetabular bone loss (Latimer and Lachiewicz 1996, Tompkins et al. 1997, Crowther and Lachiewicz 2002). There have, however, been few reports on the performance of these devices regarding wear and performance of the polyethylene bearing surface.

We evaluated/retrieved HGPI and HGPII acetabular liners that had been in situ for more than 10 years and assessed whether there was a relationship between clinical and radiographic factors, surface damage, wear, and oxidation.

## Materials and methods

### Implants and clinical information

129 HGPI and HGPII acetabular liners (in 123 patients, 69 women) with implantation times of over 10 years were retrieved between 1997 and 2010 at 4 institutions as part of a multicenter retrieval program. There were 46 HGPI liners and 83 HGPII liners. All polyethylene liners were made from GUR 4150 resin (Ticona) and were gamma-irradiated in air with (nominally) 25 kGy. The sterilization date was traceable by the manufacturer for 107 of the 129 liners. The mean shelf life (time from sterilization to implantation) was 0.96 (SD 1.10) years (range 0.06–6.7 years). For both the HGPI and HGPII liners, the inner diameter ranged from 22 mm to 32 mm and the thickness ranged from 3.3 mm to 15.3 mm (Table 1). 99 liners had a standard rim and 30 had an elevated rim (Table 1). The femoral head material was known for 122 of the 129 retrievals: cobalt chromium alloy (n = 109), zirconia ceramic (n = 10), alumina ceramic (n = 2), and titanium alloy (n = 1) (Table 1).

**Table 1.** Implant characteristics (femoral head size and material, acetabular liner thickness and rim design) for the HGPI and HGPII cohorts

	HGPI (n = 46)	HGPII (n = 83)
Head size, mm		
22	1	0
28	30	65
32	15	18
Head material		
CoCr	45	64
Zirconia	0	10
Alumina	0	2
Ti alloy	0	1
Unknown	1	6
Liner thickness, mm		
3.3	1	0
4.3	6	3
5.3	9	10
6.3	4	16
7.3	4	21
8.3	9	7
9.3	7	8
10.3	2	4
11.3	1	3
12.3	1	4
13.3	1	3
14.3	0	1
15.3	0	1
Unknown	1	2
Rim		
Standard	46	53
Elevated	0	30

The mean implantation time was 17 (SD 4.1) years (range 10–24) and 14 (SD 2.5) years (range 10–20) for the HGPI and HGPII cohorts, respectively (p < 0.001, Table 2). The mean implantation time plus shelf life (time from sterilization to implantation) was 18 (SD 4.2) years (range 11–25) and 15 (SD 2.5) years (range 11–21) for the HGPI and HGPII cohorts, respectively (p < 0.001, Table 2). The mean patient age at the time of retrieval was 65 (SD 14) years (range 41–86) and 68 (SD 16) years (range 27–91) for the HGPI and HGPII cohorts, respectively (p = 0.2, Table 2). Mean BMI at the time of retrieval was 28 (SD 5.3) and 27 (SD 4.6) for the HGPI and HGPII cohorts, respectively (range 14–44) (Table 2). For both cohorts combined, the reasons for revision were osteolysis/loosening (66%), polyethylene wear (32%), dislocation (8%), dissociation (5%), periprosthetic fracture (8%), and infection (5%) (Table 3). Preoperative diagnoses included osteoarthritis (44%), trauma (10%), avascular necrosis (12%), acetabular dysplasia (9%), and slipped capital femoral epiphysis (5%) (Table 4). 27 individuals had undergone previous revision(s) of the liner.

**Table 2.** Implantation time, patient age at revision, and patient BMI for the HGPI and HGPII cohorts

	Implantation time (years)	Implantation time + shelf time (years)	Patient age at revision (years)	BMI
HGPI	17 (4) [10–24]	18 (4) [11–25]	65 (14) [41–86]	28 (5) [14–44]
HGPII	14 (3) [10–20]	15 (3) [11–21]	68 (16) [27–92]	27 (5) [16–44]

Values are mean (SD) [range].

**Table 3.** Indications for revision for the HGPI and HGPII cohorts

Indication for revision	HGPI (n = 46)	HGPII (n = 83)
Osteolysis/Loosening	28	58
Polyethylene Wear	20	21
Dislocation	0	10
Dissociation	2	5
Periprosthetic Fracture	5	5
Infection	4	3
Other	3	5

**Table 4.** Preoperative diagnoses for the HGPI and HGPII cohorts

Preoperative diagnosis	HGPI (n = 46)	HGPII (n = 83)
Osteoarthritis	20	37
Trauma	5	8
Avascular necrosis	5	10
Acetabular dysplasia	5	7
SCFE	3	3
Other	10	14

### Analysis of surface damage

After visual examination and confirmation of the implant design, the implants were cleaned, catalogued, photodocumented, and stored either at room temperature or in a –80°C freezer. Before 2002, we did not follow the protocol recommendations of freezer storage to stop oxidative degradation of polyethylene after revision or removal surgery. The liners were assessed for damage on the articular and backside surfaces, using a semi-quantitative method developed by Hood et al. (1982) and modified for the hip. In this assessment, care was taken not to include damage that was thought to have occurred during revision or removal surgery. 98 of the 129 liners were available for analysis of surface damage. Damage scores for each surface were determined for each acetabular liner by dividing the component into 4 quadrants. Each quadrant was scored on a 0- to 3-point scale in 7 categories: pitting, scratching, burnishing, delamination, abrasion, cold flow, and embedded debris (Hood et al. 1982). A score of 0 meant no damage; a score of 1 meant damage to less than 10% of the surface area, 2 meant damage to 10–50% of the surface area, and 3 meant that more than 50% of the surface area had been damaged. The maximum possible damage score was 84. Delamination included incipient subsurface, in addition to frank delamination, and we noted whether it occurred on the liner rim or on the articular surface.

**Figure 1.**
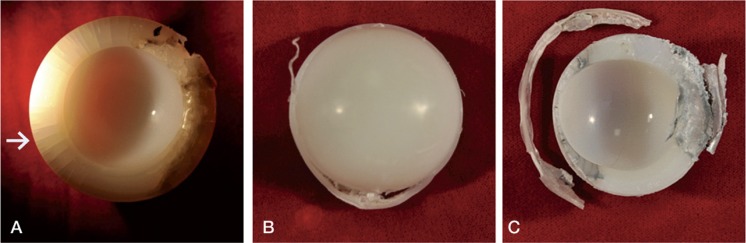
Retrieved acetabular components showing: type I subsurface cracking (HGPII) (panel A), type II fracture (HGPII) (panel B), and type III fracture (HGPII) (panel C).

In addition to compilation of scores of articular and backside surface damage, subsurface cracking and rim fracture were assessed. Liners were categorized as follows: type 1 was cracking of the subsurface only (and a percentage was used to quantify the involvement of the rim), type 2 was incomplete rim fracture (where the rim was partially separated from the liner), and type 3 was complete rim fracture (Figure 1).

### Oxidation analysis

92 liners were available for oxidation analysis (26 HGPI and 66 HGPII). Fourier transmission infrared spectroscopy (FTIR) was performed on microtomed sections of the superior articular and rim surfaces of the retrieved HGPI and HGPII liners. ASTM F2102 methodology (American Society for Testing and Materials 2006) was used to assess oxidation in the extracted polyethylene samples. Only liners that had been frozen within 6 months of revision or removal surgery were analyzed for oxidative degradation. The superior side of each liner was microtomed to yield thin sections of 200 μm. Heptane boiling was conducted on the thin sections for 6 h to extract absorbed lipids (James et al. 1993). Lipids have the same absorption wavelength as oxidation products. Failure to extract the absorbed lipids results in an artificially elevated oxidation peak. The sections were scanned through the thickness in 0.1-mm deep increments from the surface using a FTIR spectrometer with a microscope attachment. 32 independent scans with a resolution of 4 cm^-1 ^were averaged to produce an FTIR spectrum. The maximum oxidation index (OI) was calculated from the infrared spectra as the ratio of the area between the carbonyl peak centered at 1,715 cm^-1^ and the reference band at 1,370 cm^-1^. The literature suggests that ASTM OI values above 3 have a significantly detrimental effect on the mechanical properties of polyethylene (Kurtz et al. 2003, Currier et al. 2007, Kurtz 2009). The lower limit for OI is less than 1 (ASTM scale). All FTIR data were collected using a Thermo Nicolet 6700 FTIR spectroscope with a Continuum FTIR microscope attachment (Thermo Fisher Scientific, Waltham, MA).

### Linear penetration (wear) analysis

Linear femoral head penetration was assessed directly from the retrieved components. Acetabular liner thickness was measured in 3 consistent and well separated locations—each in the superior (worn) and inferior (unworn) regions—using a calibrated digital micrometer, and then averaged. The average thickness of the worn region was then subtracted from that of the unworn region to obtain the amount of penetration (Gonzalez della Valle et al. 2001). The average penetration rate of the femoral head was calculated by dividing the measured amount of penetration by the implantation time. Measurements were made by 2 independent observers. 97 of the 129 liners were available for linear penetration measurements. There was no statistically significant difference in linear penetration rate between the 2 observers, so the results were pooled.

### Radiolucency and osteolysis analysis

112 of 129 radiographs were assessed for osteolysis, interface radiolucencies, and signs of migration and loosening using the guidelines of the Hip Society (Johnston et al. 1990). 17 could not be evaluated because of unavailability or poor quality. Zonal analysis around the femoral component was recorded according to Gruen et al. (1979), and around the acetabular component according to DeLee and Charnley (1976). Osteolysis was assessed according to Engh et al. (2006).

### Statistics

Student t-tests or Mann-Whitney tests (depending on whether the data were normally distributed) were used to determine differences in patient age, BMI, shelf life, implantation time, damage scores, linear wear rate, and OI between the 2 designs. Regression analysis was used to determine correlations between damage scores and implantation time, and between damage scores and BMI. Mann-Whitney tests were used to determine differences in OI between regions or implant design. Correlations between OI and implantation time and OI and implantation time plus shelf life were assessed using Spearman’s rank correlation test. Mann-Whitney tests were also used to determine correlations between total articular surface damage scores and total backside surface damage scores of liners associated with radiographs noted to have or not have acetabular or femoral osteolysis. We also used Mann-Whitney tests to determine correlations between linear penetration rate and OI of liners associated with radiographs noted to have or not have acetabular or femoral osteolysis. In addition, we used them to determine correlations between rim OI of liners with and without fracture and subsurface cracking. Any p-value < 0.05 was considered significant. All statistical tests were performed using Minitab 15 software (Minitab Inc., State College, PA) or PASW Statistics 18.0.

### Ethical considerations

The study was approved by our institutional review board (IRB #12-00-50).

## Results

### Analysis of surface damage

The total average damage score for the articular surface was 25 (SD 7.8) (range 15–43) for the HGPI liner and 26 (SD 9.6) (range 4–54) for the HGPII liner (p = 0.6). The total average damage score on the backside surface was 22 (SD 9.0) (range 0–41) for the HGPI liner and 23 (SD 7.2) (range 8–40) for the HGPII liner (p = 0.5). Burnishing, pitting, and scratching were the most common modes of damage in both designs, on both the articular surface and the backside surface. The average articular surface and backside damage scores for each damage mode were similar for the 2 designs (Table 5). 21 of 32 HGPI liners had subsurface cracking around the rim involving up to 95% of the rim circumference and 52/66 HGPII liners had subsurface cracking involving up to 75% of the rim circumference. 8 of 32 HGPI liners and 16 of 66 HGPII liners had complete fracture of the rim. 4 of the 16 HGPII liners that had complete rim fracture had an elevated rim. There was no correlation between articular surface or backside damage scores and implantation time or BMI for either design. There was no correlation between articular surface delamination and implantation time for either design. Also, there was no correlation between total articular surface or total backside damage score and acetabular or femoral osteolysis.

**Table 5.** Mean (SD) damage scores by mode of damage to articular and backside surfaces

Damage mode	HGPI (32)	HGPII (66)	p-value
Articular surface			
Pitting	5.1 (3)	6.0 (3)	0.2
Scratching	5.4 (3)	6.1 (3)	0.3
Burnishing	7.4 (3)	6.6 (4)	0.2
Delamination	2.7 (3)	3.6 (3)	0.1
Abrasion	2.7 (3)	1.9 (2)	0.2
Cold flow	0.9 (1)	1.0 (1)	0.7
Embedded debris	1.2 (2)	1.1 (2)	0.8
Backside surface			
Pitting	4.3 (3)	4.4 (3)	0.9
Scratching	4.3 (2)	4.1 (1)	0.6
Burnishing	6.5 (5)	7.6 (4)	0.3
Abrasion	2.4 (4)	3.0 (4)	0.5
Cold flow	3.1 (3)	2.1 (2)	0.1
Embedded debris	1.0 (1)	1.5 (2)	0.1

### Oxidation analysis

**Figure 2.**
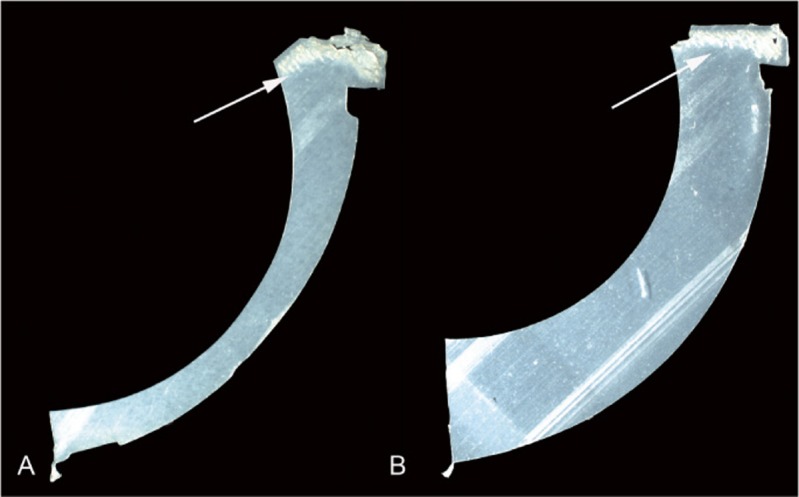
White bands seen on a micrograph of a thin section of: an HGPI that had been in situ for 22 years (panel A), and an HGPII that had been in situ for 15 years (panel B).

**Figure 3.**
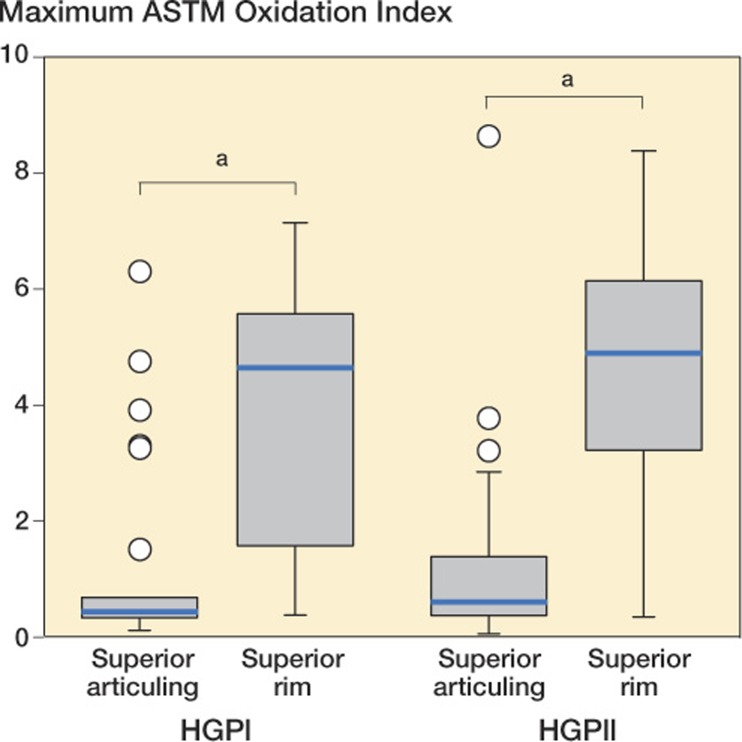
Box plots of maximum ASTM oxidation index for the superior articulating surface and the rim, for the HGPI and HGPII cohorts.

**Figure 4.**
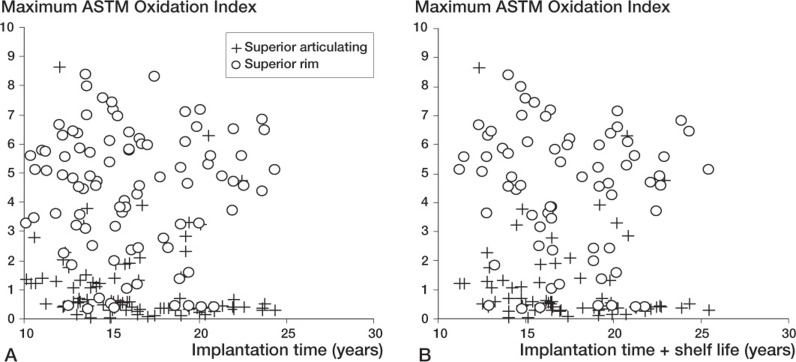
A. Maximum ASTM oxidation index vs. implantation time for the superior articulating surface and the rim for both cohorts combined. At the articulating surface, the oxidation index was negatively correlated with implantation time (Spearman’s Rho = –0.29; p = 0.005). No correlation was observed between the oxidation index at the rim and implantation time (Spearman’s Rho = –0.01; p = 0.9). B. Maximum ASTM oxidation index vs. implantation time plus shelf life for the superior articulating surface and the rim for both cohorts combined. At the articulating surface, the oxidation index was negatively correlated with implantation time plus shelf life (Spearman’s Rho = –0.24; p = 0.05). No correlation was found between the oxidation index at the rim and implantation time plus shelf life time (Spearman’s Rho = –0.099; p = 0.4).

Subsurface white bands were observed in 69 of 92 components on the superior rim following sectioning (20/26 HGPI and 49/66 HGPII) (Figure 2). White bands are evidence of variations in material density caused by oxidation. For both the HGPI and HGPII cohorts, the rim had a higher OI than the articular surface (p < 0.001 for both; Wilcoxon signed-ranks test) (Figure 3). There was no significant difference in OI at the superior articular region or the superior rim between the HGPI cohort and the HGPII cohort (p = 0.2 in both cases). The OI of the superior articular surface decreased with implantation time and with implantation time plus shelf life (Spearman’s Rho = –0.29, –0.24; p = 0.005 and p = 0.05, respectively) (Figure 4. There was no correlation between OI of the superior rim and implantation time or with implantation time plus shelf life (Spearman’s Rho = –0.014, –0.099; p = 0.9 and p = 0.4, respectively).

**Figure 5.**
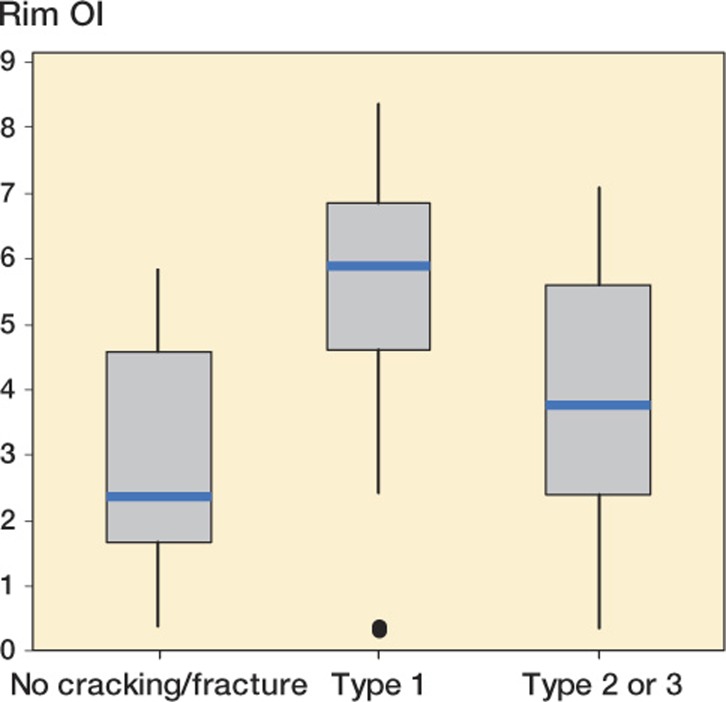
Box plots of the maximum ASTM oxidation index at the rim in liners with no subsurface cracking or fracture, subsurface rim cracking (type 1), and partial or complete rim fracture (types 2 or 3) for the 2 cohorts combined.

For the HGPI and HGPII cohorts combined, we found no statistically significant difference in OI of either the rim or the articular surface between those cases that were noted to have or not to have acetabular osteolysis or femoral osteolysis. However, the rim OI was higher in those liners with subsurface cracking than in those with either no fracture or partial or complete rim fractures (p < 0.001 in both cases) (Figure 5). There was no statistically significant difference in rim OI in those with no fracture and in those with partial or complete rim fracture.

### Linear penetration (wear) analysis

**Figure 6.**
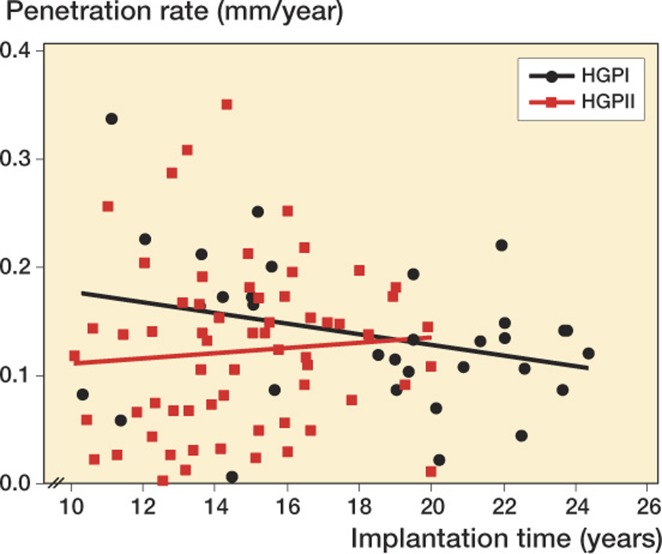
Linear penetration rate vs. implantation time (black circles: HGPI; red squares: HGPII).

The linear penetration rate was 0.14 (SD 0.07) mm/year for the HGPI liners and 0.12 (SD 0.08) mm/year for the HGPII liners (p = 0.4). The linear penetration rate decreased with time for the HGPI liners (p = 0.1) (Figure 6). There was no statistically significant difference in linear penetration rate between implants that were associated with actebular osteolysis and those that were not or between implants that were associated with femoral osteoloysis and those that were not.

### Radiolucency and osteolysis analysis

6 of the 112 femoral components showed gross femoral stem migration, which was seen on both anteroposterior and lateral radiographs. 4 of 6 were associated with radiolucent lines greater than 2 mm in thickness in one or more zones. Radiolucent lines were seen in zone 1 (21%), zone 2 (10%), zone 3 (9%), zone 5 (9%), zone 6 (8%), and zone 7 (14%). No radiolucent lines were seen in zone 4. Radiolucent lines were seen in acetabular zone 1 (27%), zone 2 (39%), and zone 3 (40%); all lines were greater than 2 mm in thickness. Osteolysis was radiographically observed around 39 of 112 femoral components (17 HGPI and 22 HGPII) and 64 of 112 acetabular components (18 HGPI and 46 HGPII).

## Discussion

Successful THA has numerous prerequisites including restoration of anatomy, durable fixation of implants to the underlying bone, and a durable, wear resistant bearing surface couple. While most previous reports of THA with the Harris-Galante Porous prosthesis and its successor, the HGPII, have focused on clinical performance in terms of fixation and loosening (Crowther and Lachiewicz 2002, Engh et al. 2004, Imai et al. 2009), we assessed the long-term outcome of the polyethylene bearings used in these devices by analysis of retrieved components obtained at the time of revision THA. We wanted to determine if there was a relationship between clinical and radiographic factors, surface damage, wear, and oxidation and if there was a difference in the implant performance or clinical performance of the HGPI and HGPII cohorts.

This study had some limitations. By their nature, implant retrieval studies are limited and imperfect in study design, in that they usually deal with analysis of devices that have been removed or revised due to clinical failure. As such, it is not possible to control for materials-processing variations that could influence the ageing of the polyethylene, including batch-to-batch variations in polymer resin or consolidation, total radiation dose, radiation dose rate, and temperature during irradiation. Even so, the analysis of retrieved implants can help track the natural history of the performance of implant materials and design features. Evaluation of long-term retrievals can be particularly valuable in this regard. In addition, the retrieved implants in this study were not handled uniformly following explantation, in that some implants were immediately frozen (to arrest ex vivo oxidation) while others were not frozen for several months. This was because the importance of immediate freezer storage on ex vivo oxidation was not recognized until after some of these implants had been revised. To account for this variation in handling procedures, only those devices that were immediately frozen were analyzed for oxidation.

Our study also had several strengths. We were able to evaluate a large number of HGPI and HGPII acetabular liners with implantation times exceeding 10 years. In addition, all polyethylene liners were made from a single resin (GUR 4150) and all were sterilized using gamma irradiation in air. These similarities facilitated comparisons between the 2 cohorts.

Not surprisingly, the polyethylene liners retrieved from acetabular components of these 2 designs performed similarly with regard to mode of damage to both the articular and backside surfaces, total damage score of both the articular and backside surfaces, and annualized linear penetration (wear) rate. For both designs, the articular surface damage scores were comparable to those of their respective backside surface damage scores; this may be due to the fact that on both surfaces the most common modes of damage were the same (e.g. pitting, scratching, and burnishing). This finding also suggests that there was some relative motion on the backside of the liner and the metal backing.

Implants of both designs also showed substantial and inhomogeneous oxidative degradation of the polyethylene liners; this has been shown to be associated with gamma irradiation in air (Kurtz et al. 2005). In addition, the GUR 4150 resin contained calcium stearate, which has been associated with fusion defects and oxidation of the polyethylene (Kurtz 2009). Notably, at a mean implantation time of 18 years for the HGPI implants and 14 years for the HGPII implants, more than two-thirds of these retrieved devices had subsurface cracking and one quarter had a complete fracture through the acetabular rim.

With both the HGPI and HGPII designs, we observed a statistically significant increase in oxidation index at the acetabular rim compared to the superior articular surface of the liners. This may be due to the greater accessibility of oxygenated body fluids to the rim, as well as the greater surface-to-volume ratio of the rim relative to the articular surface. We have previously shown that peripheral areas of the polyethylene acetabular liner (i.e. the acetabular rim) typically have a higher oxidation index and more oxidation-related degradation than those central areas under the femoral head that are shielded from physiological oxygen levels in the tissues and synovial fluid (Kurtz et al. 2006). We also found that the OI of the articular surface decreased with implantation time. This is probably related to the progressive wear of the articular surface, resulting in the removal of the most oxidized material. (However, it should be noted that when a white band is present, the maximum OI would be expected to occur in conjunction with the white band). Interestingly, we also found that the rim OI was higher for liners in which subsurface cracks were found, relative to liners showing either no fracture or partial/complete fracture. It is not unexpected that the OI at the rim of liners with subsurface cracks would be statistically significantly higher than those liners that have no cracks or fractures, because oxidation has been associated with delamination (Medel et al. 2011). It is possible that the lower OI of liners with partial or complete rim fractures relative to liners with subsurface cracks is a consequence of the fact that in these retrievals the highly oxidized portion of the rim was often lost.

Dissociation of the acetabular liner from the shell was seen in approximately 5% of patients in this study, and was always associated with a fracture of the rim of the liner. The mechanism of rim fracture of these liners appears to be a consequence of the oxidative degradation of the liners that progresses in vivo after implantation (Kurtz et al. 2006). As these liners underwent time-dependent oxidative degradation in vivo, it is likely that the rim area experienced progressive damage, with the potential for compromise of the locking mechanism and dissociation. In vivo oxidation can be expected to continue with longer implantation times; hence, the HGPI and HGPII patient populations may be at risk of an increase in late dissociation of the acetabular liner from the shell. Dissociation of the liner from the acetabular shell can result in severe damage to the titanium shell and the femoral head, giving rapidly progressive metallosis that warrants urgent revision (Werle et al. 2002, Saito et al. 2008). Under these circumstances, the locking mechanism is frequently severely damaged and a new liner must be cemented in place or the entire acetabular component must be revised.

The annualized linear penetration rates of approximately 0.12–0.14 mm/year for the HGPI and HGPII cohorts are consistent with reports of wear rates for other THA designs that used historical polyethylene (gamma radiation-sterilized in air) (Kurtz 2009). There was little relationship between the linear penetration measurements and the radiographic observations of acetabular or femoral osteolysis for either cohort. This is most likely due to heterogenous host responses to particulate wear debris, which are unique for each patient, and the recognized tendency of plain radiographs to lead to underestimation of the volume and extent of osteolysis. Previous reports have, however, indicated that a wear rate of greater than 0.1 mm/year is consistent with an increased incidence of osteolysis (Dumbleton et al. 2002).

Despite modification of the HGP locking mechanism in the HGPII design, our findings support the observation that dissociation of the liner from the acetabular shell can still occur if fracture of the rim of the liner develops due to oxidative degradation (Werle et al. 2002, Saito et al. 2008). The design of the locking mechanism of these acetabular components serves to prevent dislodgment of the liner from the shell and to limit rotation of the liner about an axis perpendicular to the opening face of the shell. Dissociation of the liner from the shell in this study appeared to happen mainly by loss of support of the rim of the liner after fracture had occurred, which allowed the liner to rotate out of the plane of the opening of the acetabular shell.

Although contemporary polyethylenes have several manufacturing processes to minimize free radical generation and reduce the likelihood of oxidative degradation, oxidation may still occur in vivo, although through a different mechanism (e.g. mechanically induced) and probably at a slower rate than for historical polyethylenes. Those designing new implants should recognize the possibility of enhanced oxidation occurring in the area of the acetabular rim and they should ensure that the integrity of the locking mechanism would not be compromised by deterioration of the material properties of the liner in the area of the rim due to oxidation, if it occurred.
